# The long-term prognosis of patients with delirium in the acute phase of stroke: PRospective Observational POLIsh Study (PROPOLIS)

**DOI:** 10.1007/s00415-019-09471-1

**Published:** 2019-07-19

**Authors:** Paulina Pasińska, Aleksander Wilk, Katarzyna Kowalska, Aleksandra Szyper-Maciejowska, Aleksandra Klimkowicz-Mrowiec

**Affiliations:** 1 Department of Neurology, University Hospital, Krakow, Poland; 2 Department of Neurology, School of Medicine, Jagiellonian University, Botaniczna 3, 31-503 Kraków, Poland; 3 Department of Neurosurgery and Neurotraumatology, University Hospital, Kraków, Poland

**Keywords:** Stroke, Delirium, Long-term prognosis, Mortality

## Abstract

**Background and purpose:**

Delirium is a very common neurobehavioral complication after stroke, but its influence on long-term outcome is not well characterized. The objective of the study was to determine the prognostic significance of delirium for functional status, nursing home admission, and mortality in a large cohort of patients with delirium in the acute phase of stroke assessed 3 and 12 months after stroke.

**Methods:**

All stroke survivors included in PROPOLIS were followed up (*n* = 682). Outcome data included: discharge destination, recurrence of stroke, cardiovascular complications, functional activity and mobility, nursing home admission, and mortality.

**Results:**

Patients with delirium were discharged to another hospital or nursing home significantly more often than those presenting without delirium. The 3- and 12-month post-stroke mortality rates were higher in delirious patients (OR 6.41 CI 3.76–10.92; *p* < 0.001 and OR 5.17 CI 3.36–7.96; *p* < 0.001). When considering 3-month mortality, higher age, modified Rankin Scale prior to admission and temperature between 1 and 3 days after admission, as well delirium, pneumonia and more severe neurological deficits on admission were independent risk factors. For 12-month mortality, the independent risk factors were higher age and modified Rankin Scale post-stroke, delirium, and history of respiratory diseases prior to stroke. Patients with delirium were more likely to live in nursing homes 3 and 12 months after stroke and were more disabled than patients without delirium.

**Conclusions:**

Delirium in acute phase of stroke negatively influences the long-term prognosis. A study addressing the effect of early recognition and treatment of identified modifiable risk factors for adverse long-term outcomes is urgently needed to decrease bad prognosis within this population.

**Electronic supplementary material:**

The online version of this article (10.1007/s00415-019-09471-1) contains supplementary material, which is available to authorized users.

## Introduction

Delirium is a common complication of stroke, with the frequency estimated to be between 10 and 48% [1]. There is abundant data linking delirium to adverse clinical outcomes in medical and surgical geriatric populations; however, post-stroke delirium remains understudied with little known about its effect on clinical outcome and long-term prognosis. Additionally, the limited available studies on long-term prognosis for patients with delirium in the acute phase of stroke show inconsistent data regarding long-term life expectancy [2–8].

Long-term medical consequences for both delirious patients and healthcare budgets are serious. Considering the inconsistent data from previous studies and the lack of data on the outcome of delirium in the acute phase of stroke in the Polish population, the aim of this single-center PRospective Observational POLIsh Study on post-stroke delirium (PROPOLIS) was to determine the prognostic significance of delirium for discharge destination, recurrence of stroke, cardiovascular events, functional status, nursing home admission, and death. This was assessed by following a large cohort of patients with stroke for 12 months.

## Materials and methods

### Population and design

**Fig. 1:**
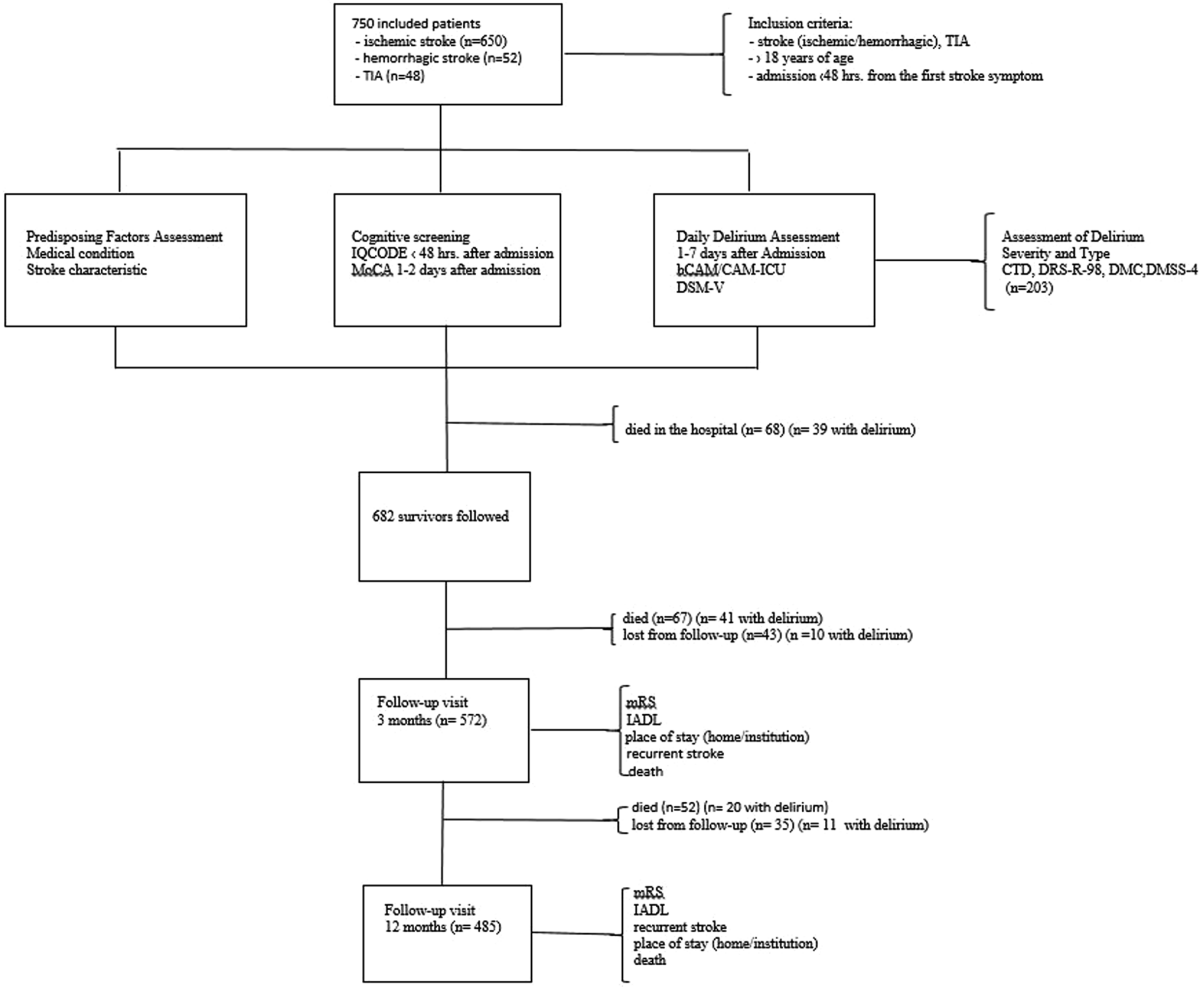
Flowchart of the study procedures

A total of 750 consecutive patients admitted to the Stroke Unit at the University Hospital in Krakow between June 2014 and March 2016, presenting with stroke (ischemic or hemorrhagic) or transient ischemic attack, and meeting the inclusion criteria for this study (patients over 18 years of age, admitted within 48 h from the first stroke symptoms, speaking Polish) were included in the assessment [9]. Figure 1 shows the study procedures.

Patients were screened for delirium every day from admission to the 7th day of hospitalization with the abbreviated version of Confusion Assessment Method (bCAM). In those with motor aphasia, or those who could not communicate for other reasons, the Intensive Care Units version (CAM-ICU) [10, 11] was adopted. To assess the severity of delirium symptoms, the Delirium Rating Scale Revised 98 [12] and Cognitive Test for Delirium [13] were applied.

A resident neurologist specially trained in delirium diagnosis was responsible for screening for delirium, and a trained psychologist was responsible for cognitive assessment. The senior neurologist/neuropsychologist was responsible for evaluating all data. The physicians rating the patients did not change during the study.

To obtain information for possible delirious symptoms during the first 24 h, a short questionnaire regarding patients’ behavior and cognitive fluctuations, was completed by ward nurses for each patient.

Diagnosis of delirium was based on clinical observation and structural assessment. The final diagnosis of delirium was based on DSM-5 [14]. For those patients who were not able to undergo cognitive evaluation, the diagnosis was based on clinical observation and DSM-5 criteria for delirium.

Data were collected regarding socio-demographic factors, smoking (current, ex-smoker, and never smoked), and comorbidities (hypertension, diabetes mellitus, atrial fibrillation, myocardial infarct, percutaneous coronary intervention, coronary artery bypass grafting, respiratory system disorders, gastrointestinal complications, liver and renal dysfunctions, genitourinary problems, past neurological history, musculoskeletal dysfunctions, and endocrine problems). The Cumulative Illness Rating Scale (CIRS) was used as a general indicator of health status [15]. To screen for pre-stroke dementia Polish version of Informant Questionnaire on Cognitive Decline in the Elderly (IQCODE) was used [16].

Both cognitive and behavior/emotional functioning were also assessed during hospital stay. For cognitive assessment, the Montreal Cognitive Assessment (MoCA) [17], Frontal Assessment Battery [18], and Cognitive Test for Delirium [13] were used between first–second day and on 7th day after admission. On admission, information was obtained from the spouse/caregiver regarding pre-stroke behavioral functioning on Neuropsychiatric Inventory [19].

All patients had neuroimaging (CT/MRI) performed at admission. The severity of clinical deficit was graded by the National Institutes of Health Stroke Scale (NIHSS) [20], also upon admission. Disability prior to admission was assessed by the modified Rankin Scale (mRS) [21]. Details of data acquisition and patients characteristics are described elsewhere [9].

### Outcome assessment

All patients dismissed from the hospital were scheduled for a follow-up visit 3 and 12 months after their stroke. Outcome assessment included discharge destination (home, rehabilitation hospital, long-term institution), occurrence of a vascular event (heart attack, any heart surgery, cardioversion, and endarterectomy), dependence (mRS), or death. Information regarding recurrence of stroke or TIA, pneumonia [22], place of stay (patient’s own home, hospital, nursing home), and the level of activities of daily leaving (I.A.D.L.) [23] was also collected.

Patients who did not attend a follow-up visit were contacted by phone. If the patient could not be interviewed, a next-of-kin was contacted and information was gathered. Mortality data were collected when a researcher was reliably informed of the death of a participant, usually by a close informant of their household.

The medical ethical committee of the Jagiellonian University approved the study. Informed written consent was provided by the patient or a caregiver.

### Statistics

All of the statistical analyses were calculated using STATISTICA for Windows version 12 (StatSoft Inc., Tulsa, Oklahoma). First, descriptive statistics were performed to obtain information about the group. Associations between 3-month and 12-month mortality and predisposing factors were found and *p* values were obtained using univariate logistic regression (Table 1). Variables significantly associated with delirium according to the univariate logistic regression were then entered into multivariable logistic regression analysis. Best fitted 3-month and 12-month mortality models were obtained using forward stepwise selection method. Final model included age and was then adjusted for sex and comorbidities (CIRS score). Kaplan–Meier curves were used to present 3-month and 12-month survival according to post-stroke delirium incidence. Gehan’s Wilcoxon tests were used to compare mortality in these groups. At the end, other outcome data were compared—categorical variables (stroke recurrence, cardiovascular complications, and discharge destination) according to delirium incidence using Chi-squared test and quantitative variables (mRS, IADL) using Mann–Whitney test. The value of *α* = 0.05 was considered as a threshold for statistical significance.

**Table 1:** Baseline characteristic of studied population

	Valid *N* at baseline	Mean (SD) or count (%)	Delirium mean (SD) count (%)	No delirium mean (SD) count (%)	Mortality 3 months post-stroke (univariate analysis *p* value)	Mortality 12 months post-stroke (univariate analysis *p* value)
Total	750					
Delirium			203 (27%)	547 (73%)	< 0.001	< 0.001
Mean CTD	136	11.2 (5.9)	11.2 (5.9)	–	0006	< 0.001
Mean DRS	203	18.2 (4.5)	18.2 (4.5)	–	< 0.001	< 0.001
Duration time	203	4.2 (2.3)	4.2 (2.3)	–	> 0.05	0.044
Mean age (SD), years	750	71.8 (13.1)	77.0 (10.7)	69.8 (13.4)	< 0.001	< 0.001
Hemorrhagic stroke	750	52 (7%)	24 (12%)	28 (5%)	> 0.05	> 0.05
Ischemic stroke	750	650 (87%)	170 (84%)	480 (88%)	> 0.05	> 0.05
TIA	750	48 (6%)	9 (4%)	39 (7%)	> 0.05	> 0.05
Gender (male) (%)	750	352 (47%)	84 (41%)	268 (49%)	> 0.05	> 0.05
Cerebellar stroke vs hemispherical/brainstem	735	73 (10%)	8 (4%)	65 (12%)	> 0.05	> 0.05
Left vs. right hemisphere	661	364 (55%)	91 (45%)	273 (50%)	> 0.05	> 0.05
Education (SD), years	658	11.4 (3.3)	10.5 (3.1)	11.7 (3.4)	> 0.05	0.045
IQCODE (SD)	610	83.3 (11.5)	86.8 (15.5)	82.0 (9.1)	< 0.001	< 0.001
The Montreal Cognitive Assessment	511	19.3 (6.8)	11.4 (6.5)	21.0 (5.5)	< 0.001	< 0.001
Hypertension	747	533 (71%)	145 (71%)	388 (71%)	> 0.05	> 0.05
Diabetes mellitus	747	203 (27%)	71 (35%)	132 (24%)	> 0.05	> 0.05
Atrial fibrillation	748	180 (24%)	76 (37%)	104 (19%)	0.014	< 0.001
Myocardial infarct	747	106 (14%)	33 (16%)	73 (13%)	> 005	> 0.05
Respiratory diseases (0–4)	747	1.0 (1.1)	1.2 (1.3)	0,.9 (1.0)	0.004	< 0.001
CIRS						
Total Score	747	9.4 (4.9)	11.0 (4.7)	8.8 (4.9)	< 0.001	< 0.001
Severity index	747	0.7 (0.4)	0.8 (0.4)	0.6 (0.4)	< 0.001	< 0.001
Comorbidity Index	747	3.5 (1.9)	4.2 (1.8)	3.3 (1.9)	< 0.001	< 0.001
Pneumonia at admission	750	72 (10%)	41 (20%)	31 (6%)	< 0.001	< 0.001
Pneumonia during hospitalization	750	44 (6%)	23 (11%)	21 (4%)	0.039	< 0.001
Urinary tract infection at admission	723	207 (29%)	85 (42%)	122 (22%)	0.001	< 0.001
Urinary tract infection during hospitalization	723	44 (6%)	13 (6%)	31 (6%)	> 0.05	> 0.05
Hospitalization (days)	750	11.1 (7.3)	14.0 (10.6)	10.0 (5.3)	> 0.05	< 0.001
Aphasia	750	255 (34%)	89 (44%)	166 (30%)	< 0.001	< 0.001
Neglect	750	94 (13%)	62 (31%)	32 (6%)	0.038	> 0.05
Vision deficits	750	298 (40%)	145 (71%)	153 (18%)	< 0.001	< 0.001
NIHSS	750	8.5 (7.3)	13.0 (6.7)	6.9 (6.8)	< 0.001	< 0.001
Pre-stroke modified Rankin Scale	749	0.7 (1.4)	1.3 (1.7)	0.5 (1.2)	< 0.001	< 0.001
mRS (7–10 day after admission)	750	2.6 (2.0)	4.1 (1.6)	2.1 (1.9)	< 0.001	< 0.001
Maximal temperature between 1 and 3 day	750	37.1 (0.7)	37.4 (0.7)	37.1 (0.7)	< 0.001	< 0.001
Maximal temperature between 4 and 7 day	726	37.0 (0.7)	37.2 (0.7)	36.9 (0.6)	< 0.001	< 0.001

Delirium Rating Scale Revised 98, Cognitive Test for Delirium IQCODE Informant Questionnaire on Cognitive Decline in the Elderly, *mRS* modified Rankin Scale, *NIHSS* National Institute of Health Stroke Scale, *CIRS* The Cumulative Illness Rating Scale

## Results

**Fig. 2:**
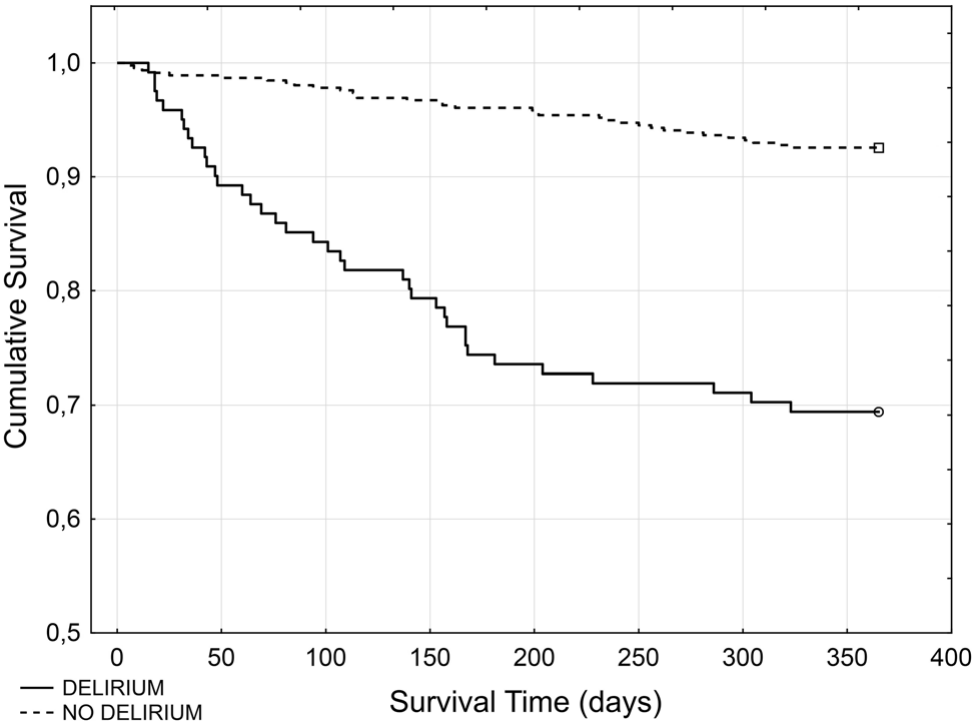
Kaplan–Meier curve demonstrating the cumulative mortality rate of patients with and without post-stroke delirium 3 and 12 months following discharge

Of the 750 patients included 682 stroke survivors were scheduled for follow-up visits (a flow chart shows the study design). From them, 67 died and 43 were lost from the follow-up 3 months post-stroke. Ultimately, patients with delirium in the acute phase of stroke experienced increased mortality within 3 months post-stroke (26.62% vs. 5.36%, Person’s *χ*^2^ 56.302 *p* < 0.001; survival comparison shown in Fig. 2—Gehan’s Wilcoxon test *p* < 0.001). The baseline characteristic of the stroke population and risk factors for death after 3 months are shown in Table 1. Patients who died were also significantly older, had more sever delirium and cognitive impairment prior to and during hospital stay, had more sever concomitant chronic diseases (in particular atrial fibrillation and diseases affecting respiratory tract) and higher temperature between days 1 and 7 of hospitalization. Death was also significantly more common in those with aphasia, neglect and vision deficit and in those more disabled on admission and on discharge.

Delirium was found to be an independent risk factor for death in univariate logistic regression analysis (OR 6.41 CI 3.76–10.92; *p* < 0.001). In the multivariable logistic regression model, delirium remained significantly associated with mortality 3 months after stroke (results are shown in Table 2).

**Table 2:** Independent risk factors for mortality 3 and 12 months post-stroke in multivariable logistic regression analysis

3-Months mortality	OR (CI)	*p*	OR (CI) adjusted*	*p*
Age	1.05 (1.02–1.08)	0.002	1.06 (1.02–1.09)	0.003
Pre-mRS	1.65 (1.37–1.99)	< 0.001	1.64 (1.35–1.99)	< 0.001
Delirium	2.90 (1.58–5.31)	0.001	2.83 (1.54–5.21)	0.001
Pneumonia on admission	2.47 (1.13–5.39)	0.023	2.23 (0.98–5.10)	0.057
NIHSS on admission	1.05 (1.01–1.10)	0.027	1.05 (1.01–1.10)	0.025
Maximal temperature between 1 and 3 day	1.59 (1.02–2.47)	0.042	1.58 (1.01–2.47)	0.043
Sex	–	–	1.63 (0.85–3.15)	0.143
CIRS	–	–	1.02 (0.96–1.09)	0.670

12-Months mortality	OR (CI)	*p*	OR (CI) adjusted*	*p*
Age	1.09 (1.06–1.12)	< 0.001	1.09 (1.06–1.13)	< 0.001
Delirium	1.75 (1.03–2.97)	0.038	1.72 (1.01–2.92)	0.044
Respiratory diseases	1.54 (1.22–1.93)	< 0.001	1.45 (1.14–1.84)	0.003
mRS between 7 and 10 day	1.89 (1.60–2.24)	< 0.001	1.91 (1.61–2.28)	< 0.001
Sex	–	–	1.61 (0.92–2.84)	0.098
CIRS	–	–	1.02 (0.97–1.08)	0.397

*mRS* modified Rankin Scale, *NIHSS* National Institute of Health Stroke Scale, *CIRS* The Cumulative Illness Rating Scale

*Adjusted for sex and comorbidities (CIRS scale)

Twelve months after their strokes, 485 patients attended follow-up visits (52 died and 35 were lost from the follow-up). Mortality 12 months post-stroke was higher in those with delirium (42.66% vs. 12.58%, Person’s *χ*^2^ 62.406; *p* < 0.001; survival comparison shown in Fig. 2—Gehan’s Wilcoxon test *p* < 0.001). Patients who died were also older, had more severe delirium and cognitive impairment prior to and during hospital stay, were less educated, had longer hospitalization, more severe concomitant chronic diseases (in particular atrial fibrillation and diseases affecting respiratory tract) and higher temperature between 1 and 7 day of hospitalization. Death was also more often in those with aphasia, vision deficit and in those more disabled on admission and on discharge.

Delirium was found to be an independent risk factor for 12 months mortality in univariate logistic regression analysis (OR 5.17 CI 3.36–7.96; *p* < 0.001). In multivariable logistic regression model, delirium remained significantly associated with 12-months post-stroke mortality (results are shown in Table 2).

Patients with delirium were also more disabled and reported a larger impairment on daily activities than those without delirium 3 and 12 months post stroke (Table 3 shows these results).

**Table 3:** Functional impairment in patients with and without post-stroke delirium 3 and 12 months after stroke

3-Months post-stroke	Patients without delirium		Patients with delirium		*p*
	median	IQR	median	IQR	
mRS	1	3	4	2	< 0.001
IADL	10	10	27	12	< 0.001

12-Months post-stroke	Patients without delirium		Patients with delirium		*p*
	Median	IQR	Median	IQR	
mRS	1	2	3	3	< 0.001
IADL	9	9	27	15	< 0.001

*mRS* modified Rankin Scale, *IADL* Instrumental Activities of Daily Leaving

Three months post stroke, patients with delirium were less often at home and were more often hospitalized or placed in a nursing home compared to patients without delirium (47.32; 33.04; 19,64% vs. 74.56; 22.81; 2.63%, respectively). The Chi-square test was highly significant (*p* < 0.001).

The results regarding living situations were consistent in the second follow-up meeting. Twelve months post stroke, patients with delirium were at home or hospitalized less often, and were placed more often in nursing home than patients without delirium (64.56; 0.00; 35.44% vs. 93.29; 0.48; 6.24%, respectively). The Chi-square test was highly significant (*p* < 0.001). In multivariable logistic regression model adjusted for NIHSS, CIRS, pre-mRS, age, delirium remained significantly associated with higher disability and impairment on daily activities at 3 and 12-months post-stroke (Supplementary materials).

We did not find a significant association between delirium and recurrence of stroke or cardiovascular complications 3 and 12 months after stroke.

## Discussion

The results of this study showed that delirium is independently associated with a poorer prognosis both 3 and 12 months after stroke, including greater functional disability, higher rates of admission to long-term institutionalized facilities, and greater mortality, when compared to delirium-free patients.

This study identified a number of risk factors for post-stroke mortality. Within these factors, increased temperature in acute phase of stroke and delirium were the only modifiable risk factors.

Our study confirmed some but not all of previously reported results. In a study by Dostovic et al. [4] that followed 100 patients with delirium and 100 controls with stroke, delirious patients had a significantly higher mortality and a greater degree of functional disability in the first year after ischemic stroke. Sheng et al. [2] followed 140 patients with stroke and found that delirium predicted 6 and 12 months mortality, disability and institutionalization. Both Henon et al. [6], who followed 202 patients with stroke, and McManus et al. [5], who recruited 82 patients, did not find increased mortality at 6 and 12 months, respectively. Miu et al. [3] included 314 patients with stroke and followed them for 1 year. This study found that patients with delirium had more functional disability and more often were placed in nursing homes at discharge. Mortality 6 and 12 months after stroke was higher in patients with delirium but the differences were not significant. Oldenbeuving et al. [7] who followed 527 patients with stroke found that delirium was associated with lower functional outcome and higher mortality 1-month post stroke. Qu et al. [8] who observed 261 patients with ischemic stroke for 6 months found that delirium was associated with worse functional outcome after 3 months.

Stroke patients often suffer from temperature elevations without an identifiable infection; possibly endogenous fever due to direct stroke effects on the brain [24]. We identified that increased temperature between first and third-day of the hospital stay independently increased mortality 3 months after stroke. The data about temperature management in acute stroke are inconclusive [25]. Our data provide support for trials testing temperature lowering in acute phase of stroke as a preventing strategy for unfavorable long-term outcome.

Results of our study confirm the evidence from medical literature that delirium predicts a poor outcome [26]. Previous studies on the long-term outcome of post-stroke delirium were conducted on small cohorts. The large number of consecutive patients with stroke is a strength of our study—there is no other prospective study on post-stroke delirium with such a big cohort. The other strength of our study was the constant observation of patients by medical personnel and careful, formal assessment of delirium conducted every day. For delirium screening we used bCAM for verbal patients or CAM-ICU for patients who could not speak but were able to communicate in a non-verbal way. Both methods have high sensitivity and specificity and are easy to administer [10, 11]. The same assessor administered the scale from day 1 to day 7, thus making the bias of inter-observer variation minimal. All this allowed for the identification of all delirium cases, even those lasting briefly.

This study included patients of a wide age rage. Enrollment of younger patients might decrease the percentage of cases with post-stroke delirium. However, the mean age of the cohort was high and similar to other studies, therefore, we do not think that including younger patients would cause a bias. The number of patients lost from follow-up was small.

A limitation of our study is that the incidence of delirium in the acute phase of stroke in our sample might be underestimated due to an observation period restricted to only 7 days. This is the median duration of hospital-stay in Krakow’s stroke unit. Therefore, those with delayed onset delirium were missed.

## Conclusions

Delirium is a common complication of stroke and its long-term consequences are very serious. Predisposing factors for its development were previously identified for the Polish population [7]. Unfavorable outcomes of patients with delirium in the acute phase of stroke emphasize the need of better awareness of this common stroke complication. As a matter of its course, data shows delirium is strongly associated with additional healthcare costs [27]. Bearing this in mind, results of our study support the need for further studies addressing the influence of early recognition and treatment of delirium in the acute phase of stroke as well as testing lowering temperature to decrease later post-stroke mortality and to decrease adverse long-term outcomes within this population, also to better appraise the associated healthcare burden.

## Electronic supplementary material

Below is the link to the electronic supplementary material.

Supplementary file1 (DOCX 13 kb)
