# Supplementary material for: The long-term prognosis of patients with delirium in the acute phase of stroke: PRospective Observational POLIsh Study (PROPOLIS)
Source: J Neurol. 2019 Jul 19;266(11):2710–7. doi: 10.1007/s00415-019-09471-1 (PMC6803586; doi:10.1007/s00415-019-09471-1)
Supplement: Supplementary file 1 — Supplementary file1 (DOCX 13 kb) [file 415_2019_9471_MOESM1_ESM.docx]

Table 4. Presence of delirium is independently associated with mRS and IADL score after 3 and 12 months post-stroke.

| Multiple regression models  (delirium adjusted for CIRS, NIHSS, pre-mRS and age)  p values | | | | |
| --- | --- | --- | --- | --- |
|  | 3 months | | 12 months | |
|  | mRS | IADL | mRS | IADL |
| Delirium | <0.001 | <0.001 | <0.001 | <0.001 |
| CIRS  Total score | 0.002 | 0.013 | 0.001 | <0.001 |
| NIHSS at admission | <0.001 | <0.001 | <0.001 | <0.001 |
| Pre-mRS | <0.001 | <0.001 | <0.001 | <0.001 |
| Age | <0.001 | <0.001 | <0.001 | <0.001 |
| Regression model | <0.001 | <0.001 | <0.001 | <0.001 |

The long-term prognosis of patients with delirium in the acute phase of stroke. PRospective Observational POLIsh Study (PROPOLIS). Journal of Neurology. Paulina Pasinska.

Corresponding author: Aleksandra Klimkowicz-Mrowiec,

Department of Neurology, Jagiellonian University, School of Medicine, Krakow, Poland

e-mail: [Aleksandra.Klimkowicz@mp.pl](mailto:Aleksandra.Klimkowicz@mp.pl)
